# Optimal control and analysis of two-color genomotyping experiments using bacterial multistrain arrays

**DOI:** 10.1186/1471-2164-9-230

**Published:** 2008-05-19

**Authors:** Francisco R Pinto, Sandra I Aguiar, J Melo-Cristino, Mário Ramirez

**Affiliations:** 1 Instituto de Microbiologia, Instituto de Medicina Molecular, Faculdade de Medicina, Av. Professor Egas Moniz, 1649-028 Lisboa, Portugal

## Abstract

**Background:**

Microarray comparative genomic hybridization (aCGH) evaluates the distribution of genes of sequenced bacterial strains among unsequenced strains of the same or related species. As genomic sequences from multiple strains of the same species become available, multistrain microarrays are designed, containing spots for every unique gene in all sequenced strains. To perform two-color aCGH experiments with multistrain microarrays, the choice of control sample can be the genomic DNA of one strain or a mixture of all the strains used in the array design. This important problem has no universally accepted solution.

**Results:**

We performed a comparative study of the two control sample options with a *Streptococcus pneumoniae *microarray designed with three fully sequenced strains. We separately hybridized two of these strains (R6 and G54) as test samples using the third strain alone (TIGR4) or a mixture of the three strains as control. We show that for both types of control it is advantageous to analyze spots in separate sets according to their expected control channel signal (5–15% AUC increase). Following this analysis, the use of a mix control leads to higher accuracies (5% increase). This enhanced performance is due to gains in sensitivity (21% increase, p = 0.001) that compensate minor losses in specificity (5% decrease, p = 0.014).

**Conclusion:**

The use of a single strain control increases the error rate in genes that are part of the accessory genome, where more variation across unsequenced strains is expected, further justifying the use of the mix control.

## Background

Microarray technology is commonly used to monitor gene expression profiles. However, microarrays have also become an established technique in bacterial comparative genomics, in which the assessment of gene inventories is made at the genomic DNA level. This technique is sometimes referred to as microarray genomotyping [1,2] but also as array based comparative genomic hybridizations (aCGH) [3,4]. Until recently it was widely assumed that adaptive evolution in bacteria occurred mainly by mutation and restricted recombination among members of the same species [5]. However, the availability of fully sequenced prokaryotic genomes changed this view since they provided ample evidence for extensive lateral gene transfer and it became clear that this was a major source of variation and fundamental to understand adaptive evolution in prokaryotes [6]. The ability to compare microbial genomes using microarrays has been used in evolutionary studies to help define microbial species, to explore the genetic diversity within individual species, to study the evolutionary history of bacterial adaptations and to identify genes potentially associated with virulence, transmission or host specificity of a given microorganism [7-9]. These studies led to the concept of a bacterial pan-genome [10] consisting of the global gene pool of a given bacterial species, that could greatly exceed the gene repertoire of any individual strain [11]. This repertoire includes the core genome, defined as the pool of genes shared by all strains of a given species, and the dispensable genome constituted by genes present in some, but not all, strains of the same species [11]. The realization that the presence of certain genes could be variable or ubiquitous among members of a same species predates the genomics era. Campbell in an early review [12], identified "euchromosomal" and "accessory" DNA and discussed the importance of gene transfer in shaping the latter. Depending on the question addressed, one may be more concerned with the presence of genes characterizing the core genome, for instance to define microbial species, or with the complement of the dispensable or accessory genome carried by particular groups of strains. Since a large number of virulence factors are carried by mobile genetic elements or are otherwise members of the accessory genome, the studies attempting to identify associations between genetic complement and virulence naturally focus on the genes not shared by all strains [7,9].

Microarrays used in aCGH experiments are frequently constructed based on the known genome sequence of one strain, which is used as a control in each experiment. Genomic DNAs of the control and test strains are labeled with two fluorochromes and hybridized onto the microarray, which has spots identifying all genes of the reference genome. After scanning and image analysis, the logarithm of the ratio (log-ratio or LR) of both fluorescent signals (test over control) is computed for each spot. Genes present in both genomes are expected to give a clear fluorescence signal in both channels (LR close to 0), whereas genes absent in the test sample will only have a signal in the control channel (negative LR) [1].

If several strains of a given species have been sequenced, then it is possible to design microarrays with reporter elements representing more than just the genes in a single reference strain. The genomes of all sequenced strains can be used in microarray design. As more and more bacterial strains are sequenced, there is a clear trend towards the use of multistrain arrays [4,7,9]. In these cases, if only one of the sequenced strains is used as a control, in addition to detecting the presence or absence of genes found in the control, a multistrain array can now measure additions, *i.e*., those genes that are present in the test but absent in the control. The additions have positive LR values [2]. Alternatively, all strains used in microarray design can be used as control if an equimolar mix of their genomic DNA is labeled with the reference fluorochrome and hybridized together with the test. This mixed control provides at least a partial signal in all spots, in contrast to the single strain control option.

After surveying published studies with multistrain arrays, it is clear that there is no universally accepted standard of which control to use in these experiments. Some studies have used the single strain control [7-9,13,14] while others adopted the mix control approach [15-17]. The former frequently argue that a multistrain mix control, while generating a control signal for every spot on the microarray, leads to an increased signal intensity of core genome spots in the control channel and complicates data analysis based on ratios [7-9,13,14]. In spite of the multiple arguments invoked to support each of the approaches, we found that a study objectively comparing aCGH performance of the single strain versus the mix control approach was lacking.

To fill in this gap, we performed a comparative study with a multistrain array for *Streptococcus pneumoniae*, covering the genomes of three sequenced strains: TIGR4 (T) [18], R6 (R) [19] and G54 (G) [20]. We used two of these strains as tests (R and G) and hybridized them with two different controls: a single strain (T) or an equimolar mix of the three strains represented in the array (T+R+G).

As the true lists of absent genes for each strain where known *a priori *in our experiments, we could evaluate the discriminatory power of a given rule to call a spot present or absent. Several statistical methods have been proposed to define this rule specially adequate to bacterial aCGH experiments [2,4,21,22]. Independently of the chosen method, the quality of the results is intrinsically related to the discriminatory power of the LR distribution. In principle, differences found at the level of the LR distribution will be propagated through the results of every aCGH analysis method. Therefore, to better evaluate the impact of the choice of control we decided to evaluate LR distributions directly, through Receiver Operating Characteristics (ROC) analysis [23]. Using this analysis, the best LR threshold (above which spots are called present) was defined and characterized by its accuracy, sensitivity and specificity. We then explored the differences in these evaluation parameters attributable to the choice of control sample. Our results show that the use of the mix control approach provide more discriminatory LR distributions, and, consequently, less errors in the classification of genes as present or absent in the studied strain.

## Results

### Expected values and empirical distribution of LR

Since the strains hybridized as test samples in our experiments have their genomes fully sequenced, we know *a priori *which spots should identify genes as present or absent. These expected results are compiled in Table 1. Following the accepted methodologies in processing the results of two-color microarray experiments, the output variable of analysis is the ratio of the signal intensity of the test channel by the signal intensity of the control channel. For simplicity, the expected signal intensity of single fluorescence channels indicated in Table 1 was assumed to take only the values 0, 1, 2 or 3. In single strain control experiments, when a spot identifies a gene present both in the test sample and in the control, both channels have maximum signal intensity, which is 3, and a resulting signal ratio of 1. If the spot identifies a gene present in the test but not in the control, the test channel and control channel will have intensities of 3 and 0, respectively. The resulting signal ratio would tend to infinity in the absence of random noise in the control channel. Still, the resulting signal ratio will be larger as compared with other spot sets. In the reverse situation, when the spot is absent in the test but present in the control, the test channel and control channel will have intensities of 0 and 3, respectively. The signal ratio is expected to be 0, and once again, due to random noise, exact zeros will not be observed, resulting in small ratio values instead.

**Table 1:** Correct calls (identification as absent or present gene) and expected test/control signal ratios for the four types of hybridization performed.

		Expected signal ratio					
		R6			G54		

Spots specific for:	Number of unique spots	Call	vs T4	vs Mix	Call	vs T4	vs Mix

T4 (T)	374	A	0/3	0/1	A	0/3	0/1
R6 (R)	230	P	3/0	3/1	A	1	0/1
G54 (G)	258	A	1	0/1	P	3/0	3/1
T4, R6 (TR)	209	P	1	3/2	A	0/3	0/2
T4, G54 (TG)	125	A	0/3	0/2	P	1	3/2
R6, G54 (RG)	113	P	3/0	3/2	P	3/0	3/2
T4, R6, G54 (TRG)	1737	P	1	1	P	1	1

If a mixture of the 3 strains represented in the array is used as control, the relative intensity of the signal in the control channel can take the value of 1 if the gene identified by the spot is present in a single strain (T, R and G spots), 2 if the gene is present in two of the strains (TR, TG and RG spots) and 3 when the gene is shared by all three strains (TRG spots). If the spot identifies a gene present in the test sample, the resulting signal ratios will be 3, 3/2 or 1, respectively. If the spot identifies a gene absent in the test strain, all the signal ratios are expected to be 0, in the absence of random noise. In practice, the three spot classes described above will produce decreasing ratio values, represented in Table 1 as 0/1, 0/2 and 0/3, respectively.

The main problem of using the strain with the larger number of genes spotted in the array as control (T) is readily detected. There are three spot classes with an expected ratio of 1, two of which have a present call while the other should lead to an absent call. In the two classes with a present call, the unity ratio results from maximal signal intensity in both test and control, while for the absent spot class, both channels should produce a null signal. In expression arrays this would not be a problem as all three classes contribute to the bulk of non-differentially expressed genes and consequently, is of no interest to the researcher. Contrastingly, in aCGH experiments it is crucial to differentiate these two outputs, the absent and the present call. One possible solution for this problem is to analyze the spots with oligos specific for T (T, TR, TG and TRG spots) separately from the ones with oligos not hybridizing with T genes.

But Table 1 also suggests another way to circumvent this problem by using the mixture of the three reference genomes (T+R+G) as control. In these hybridizations all the spot classes with absent calls have expected signal ratios lower than 1. Using the mix control produces five different expected ratio values, with three possible levels of control signal. We then expect to improve the detection of absent/present genes by performing the analysis separately for spots that are specific for one control strain (T, R and G spots), two control strains (TR, TG and RG spots) and all control strains (TRG spots).

**Figure 1:**
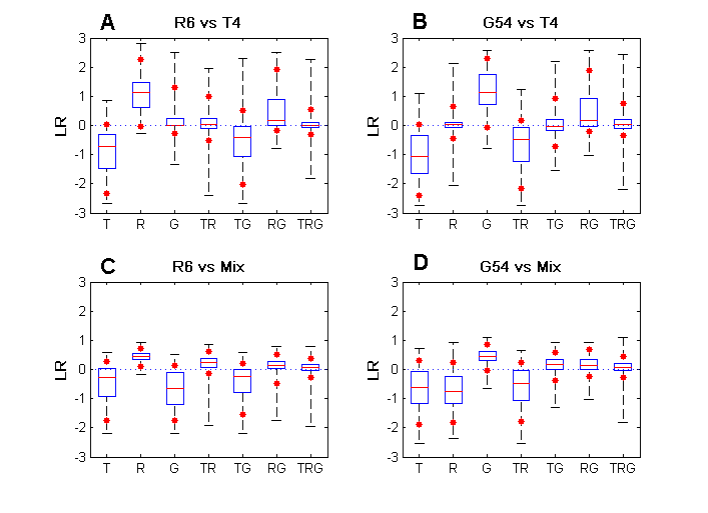
**Boxplot of LR values for different spot classes.** The limits of the central box represent the 1^st ^and 3^rd ^quartile. The line in the middle of the box marks the median. The lines in the ends of vertical broken lines represent minimum and maximum LR values observed. The stars along the broken lines indicate the 5^th ^and 95^th ^percentiles.

Figure 1 presents the actual distribution of LR values (base 10) for the distinct hybridizations performed. Each panel is the combined distribution of four replicates. Median values are in good agreement with respect to the logarithm of the expected values of Table 1. Hybridizations with T control for spot sets with an expected LR greater than 0 appear to produce LR distributions with a median more distant from 0 but with higher amplitude (Panels A and B) as compared with the same sets in the mix control experiments (Panels C and D). In the hybridizations with a mix control, for the spot sets that are expected to generate a positive LR, the dispersion is reduced as compared with the spot sets in which the test is absent (Panels C and D). In the former distributions, both test and control have signals above noise levels, while for the latter the signal of the test is just random noise, contributing to a higher LR variability. These random noise signals are also the reason for higher dispersion in LR distributions for hybridizations with the T control in spots where either the test or the control is not specifically recognized by the spots.

### Threshold independent analysis

**Figure 2:**
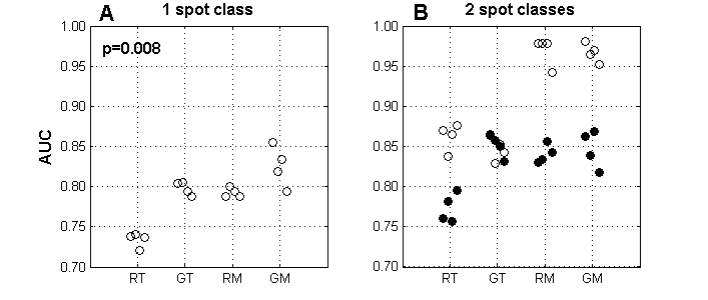
**Threshold independent analysis.** Each circle represents the area under the ROC curve (AUC) for one array. Labels in the x axis identify the hybridization: RT – R6 (test) with TIGR4 control, GT – G54 with TIGR4 control, RM – R6 with mix control and GM - G54 with mix control. Panel A: Open circles represent the values obtained by analyzing together all the spots in the array. Panel B: For RT and GT hybridizations, open circles represent the AUC values for the set of T, TR and TG spots, filled circles represent the AUC values for the set of R, G and RG spots; for RM and GM hybridizations, open circles represent AUC values for the T, R and G spots, filled circles represent the AUC values for TR, TG and RG spots.

To evaluate the discriminatory power of LR distributions without having to specify an exact threshold value, we compared de AUC of ROC plots for the hybridizations with different controls, analyzing either all spots together or divided into two mutually exclusive classes. The AUC values have a useful quantitative meaning: if one takes two spots, one with a true present call and another with a true absent call, the AUC is the probability that the LR value of the first spot is higher than that of the second spot [23]. Analyzing all spots together (Figure 2, panel A) we detected a significant increase in AUC if the mix control is used (p = 0.008). Carrying out two separate analyses according to spot class (Figure 2, panel B), we obtain a higher discriminatory power for spots recognizing T genes, when using a T control. In hybridizations with mix control, the spots specific for one strain perform better than the ones that are specific for two strains. Still, the latter have a discriminatory power equivalent to the best performing spots in hybridizations with a T control.

### Threshold dependent analysis

**Figure 3:**
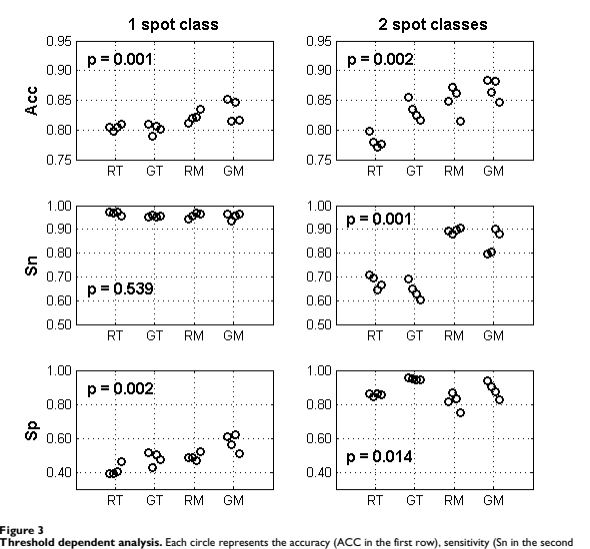
**Threshold dependent analysis.** Each circle represents the accuracy (ACC in the first row), sensitivity (Sn in the second row) or specificity (Sp in the third row) for each array using the optimal LR thresholds that maximized the accuracy values. Actual optimal threshold values are given in a supplementary table [see Additional file 1]. Labels in the x axis identify the hybridization: RT – R6 (test) with TIGR4 control, GT – G54 with TIGR4 control, RM – R6 with mix control and GM - G54 with mix control. In the left column, values were obtained analyzing together all the spots in the array. In the right column, values were obtained analyzing separately two classes of spots according to the expected control signal and excluding the TRG set of spots, as detailed in the methods section. This exclusion precludes the direct comparison of the numeric values between the left and right column. However, the removed TRG spots are expected to have similar quality performances with both types of control and should mainly increase true present call, which has a positive impact on sensitivity and accuracy values.

We determined the LR threshold that provided the best accuracy in gene presence/absence detection in each of the 16 hybridizations. Figure 3 shows the maximum accuracy values obtained, and the corresponding sensitivities and specificities. Absolute values in the left column should not be directly compared with the values in the right column, as they do not correspond to the same set of spots because the TRG spot set is not included (see methods) when spots are analyzed in classes (right column). In an hypothetical scenario with an unknown strain as test sample, inclusion of the TRG spots would mainly increase true present calls, which would increase the overall accuracy and sensitivity values. Again, we show that using the mix control there is a significant increase in accuracy, either analyzing all spots together (p = 0.001) or in two groups (p = 0.002). In the first case, the improved accuracy is due to a significantly higher specificity (p = 0.002), meaning that the mix control originates less false present calls. On average, using the mix control reduces the number of erroneous calls by 106 per array (99 false present calls and 7 false absent calls), which corresponds to an improvement of specificity of 12.5% and does not change significantly the sensitivity.

When spots are divided in two classes, the lower accuracy associated with the use of the T control is instead due to lower sensitivity. Here, the use of the mix control produces less 68 erroneous calls per array (resulting from an increase of 41 false present calls and a decrease of 110 false absent calls), which corresponds to an improvement of sensitivity of 21% (p = 0.001) and a decrease of 5% in specificity (p = 0.014).

## Discussion

A common argument against the use of the mix control is the increase in the complexity of the data analysis due to a higher number of expected signal classes that need to be analyzed separately [8]. By inspecting Table 1 it was already apparent that even with a single strain control, it is advantageous to analyze separately the spots that recognize specifically the control strain from the ones that do not. If such separate analysis were not performed, spot types with correct present and absent calls would be grouped in a class producing the same expected ratio. The improved performance of analyzing the data separated into spot classes, instead of analyzing all spots together, is apparent in Figure 2, where for both types of control AUC values are consistently higher when spots are analyzed separately. Although the advantages of performing an analysis by spot classes were not formally explored, a few previous studies using multistrain arrays with a single strain control have opted for defining two thresholds for spots present or absent in the control [9] or running the analysis algorithm twice, inverting the LR values [8].

Since there is no universal consensus on analyzing all spots together or splitting them into classes according to the expected signal in the control channel, we compared control performances in each of these situations. For both of these approaches the use of mix control led to better results, measured by increased discriminatory power of LR distributions (Figure 2) and accuracy (top row in Figure 3), although these were due to the decrease of different types of error.

When all spots are analyzed together, the mix control is responsible for higher specificity, without loss of sensitivity. In the hybridizations with the T control, the increased false positives are mainly G spots, when the test sample was R, or R spots when the test sample was G. These spots have an expected ratio of 1, which is also the expected ratio of the more abundant spot type TRG that will have more weight in defining the value of the optimal threshold. A good threshold for correctly identifying the present TRG spots is naturally a bad threshold for identifying the absent G spots (or R spots, according to the test strain), whose LR distributions are almost completely overlapping (Figure 1).

If spots are analyzed in different classes, the mix control is associated with a gain in sensitivity higher than the loss in specificity. Most of the divergence in the results of each control option is related with spots that recognize genes in the genome of two strains. In hybridizations with both types of control, the LR distributions have greater overlaps among spot types that are specific for two reference strains as compared with spot types that are specific for just one strain (Figure 1), which could be related to the fact that oligos designed for multiple strains can more easily present lower identities with their targets than oligos designed for ORFs present in just one strain. Hybridizations with the T control produce less false present calls while arrays with mix control have less false absent calls. To obtain further insights into this behavior we looked at the individual sets of spots that are in each class analyzed separately.

In hybridizations with the T control one optimal threshold is chosen from the LR values of the spot class recognizing T genes (T, TR and TG spots) while another threshold is adopted for the spot class not recognizing T genes (R, G and RG spots). This division joins in the same class sets of spots specific for one and two reference strains, creating an extensive overlap between LR distributions of present and absent genes. In the T recognizing class, the present set (TR or TG according to the test sample) is mixed with the more abundant absent sets, especially with the one that is specific for two reference strains (Figure 1), originating more false absent calls. In the class that does not target T genes, the present set that is specific for one strain only is well separated from the absent set (that is also specific for one strain only). Most errors come from the present RG spot set that overlaps significantly with the absent set that is centered on an LR value of 0. Hence, by producing more false negatives (false absent calls) than false positives (false present calls), both sets contribute to the higher specificity associated with hybridizations using the T control.

For hybridizations using the mix control the two classes of spots analyzed separately were the spot class recognizing genes present in one strain only (T, R and G spots) and the spot class identifying genes present in two strains (TR, TG and RG spots). In contrast with the classes analyzed with the T control, this division separates into a single class the spots that are specific for genes present in only one reference strain. As stated previously, among these spot sets, absent and present genes are well segregated and, consequently, the classification in this class produces few errors. The threshold value of the second class is mainly conditioned by present spots (TR or TG, according to the test sample, and RG) because they are more abundant. Consequently a higher error rate is obtained in the absent spots (TG or TR, according to the test sample), producing more false present calls. Notwithstanding, the error rates in the present sets are much lower than the ones observed for hybridizations with the T control, comparatively producing significantly less false absent calls, contributing to the higher sensitivity obtained using the mix control.

## Conclusion

The overall data favors the use of the mix control in multistrain microarray CGH experiments. Additionally, the lower accuracies described for hybridizations with the single strain control are associated with genes that are already known not to be part of the core genome of the species since they are not shared by all strains used in designing the array. Genes represented in TRG spots, that include those defining the core genome and a fraction of those included in the accessory genome, were not analyzed because, as stated in the methods section, they should be analyzed separately when the mix control is used. Since all these spots are known to be present, the methodology used to evaluate the discriminatory power of LR distributions does not apply. Still, we expect both control choices to behave similarly for TRG spots because the control channel in both approaches has the same expected signal intensity (Table 1) and therefore the resulting LR distributions for these spots are similar in terms of location and, in comparison with other spot classes, present lower dispersion (Figure 1). The remaining classes considered included genes found in a single strain or in two of the strains used to design the array, and therefore represent the accessory or dispensable genome. More variation between strains of the same species is expected for these genes that were specifically analyzed in this work, reinforcing the importance of the reported better performance associated with the use of the mix control. From a biological standpoint, the identification of particular ecotypes or variants with enhanced virulence among strains of the same species, are examples of studies that rely on the accurate detection of the presence of genes that are part of the accessory genome and these would benefit greatly from a mixed control approach. Since single strain controls are frequently used in these studies [9,14], this may lead to the incorrect identification of the absence of genes associated with the accessory genome and may contribute to the variable success of this technique in identifying different groups of strains within the same species.

Although we believe our results to be generally applicable to all multistrain microarrays used in aCGH, it is important to note that the magnitude of the observed effects might differ according to the array technology, quality of oligonucleotide design and the relative number of spots that are specific for only one or a subset of the strains used in the array design. Arrays with cDNA probes, for example, are expected to have similar sensitivity to 70 mer probes but lower specificity, which can lead to more cross-hybridization. Additionally, depending on the degree of sequence divergence between the strains used in the array design, probes targeting ORFs present in multiple strains can have lower sensitivity, lower specificity or both. In fact, the oligos in our arrays can have different sensitivities and specificities when hybridizing with the R6 or G54 strains, respectively. Additionnally, the G54 strain has more genes with spots specific for one strain only (more G spots than R spots) and less genes targeted by spots recognizing two strains (more TR spots than TG spots, Table 1), which according to the previous discussion should improve the quality of G54 results. Notwithstanding, our analysis shows that, independently of these differences, we can say that using a single strain as control will lead to higher error rates for spots that recognize genes that are not present in all reference strains. Moreover, if the mix control is used and spots are analyzed in separate classes higher sensitivities are achieved, meaning that less false absent call are produced and a higher fraction of the truly present genes are recovered from the analysis. Snipen and colleagues discuss that in aCGH experiments, the study focus of the researcher can be primarily to identify divergent or absent genes or, on the other hand, the focus can be on the present genes, for example, when estimating the conserved core genome over all strains [4]. In the first situation, it may be more important to require higher sensitivities, that is, less false absent calls, while in the second situation specificity may be more relevant, attempting to avoid false present calls. In published studies the first situation is more common, which led Repsilber and colleagues to state that in a typical genomotyping experiment high sensitivities, around 0.9, are desirable to prevent high rates of false absent calls [1]. We have shown here, that those levels are attainable for multistrain microarrays if a mix control is applied and spots are analyzed in two different classes. If spots are analyzed in a single class, high sensitivities are also observed, but accompanied by undesirable very low specificities.

If the main objective is to define a core genome and high specificity is required, analyzing spots in separate classes is mandatory whichever control is used. Again, the mix control provides the best-balanced performance since the use of the single strain control, although associated with slightly better specificities, presents substantial losses in sensitivity.

Independently of the method chosen for the analysis of aCGH data, a suboptimal choice of control sample may compromise the quality of the final results as well as the possibility to compare different studies. Our study thus provides an important source of information to support the design of bacterial genomotyping experiments with multistrain microarrays.

## Methods

### Strain growth and DNA preparation

Strains included in the present study were R6 (kindly provided by A. Tomasz), TIGR4 (obtained from the American Type Culture Collection) and G54 (kindly provided by J.F. Garcia-Bustos). Strains were grown overnight on blood agar plates at 37°C in 5% CO_2_. A few colonies were inoculated in 6 ml of Brain Heart Infusion medium and the culture was incubated at 37°C for approximately 6 h. Total DNA extraction was performed using the CTAB protocol [24].

### Genome Labeling and Microarray processing

Microarray experiments were performed using whole-genome *S. pneumoniae *oligonucleotide microarrays (version 5) obtained from the Pathogen Functional Genomic Resource center at The Institute for Genomic Research (TIGR) [25].

Briefly, the microarray contains 3425 oligos (70 mers) replicated four times targeting *S. pneumoniae *open reading frames (ORFs) and 10 amplicons and 500 oligos targeting ORFs of *Arabidopsis thaliana *as a negative control. 2445 of the *S. pneumoniae *oligos target genes present in the TIGR4 genome, 2289 in the R6 genome and 2233 in the G54 genome. Oligos were designed based on ORF sequences across all the three genomes simultaneously, attempting to provide coverage for every non-redundant ORF present in the three genomic sequences. There are oligos that bind specifically to ORFs present in two or in all of the three genomes (T, R and G). According to their specificity to one, two or three of the reference genomes, spots (or oligos) were classified in 7 sets, listed in Table 1. Annotation files provided by The Pathogen Functional Genomic Resource Center [26] contain the results of BLASTN searches of each designed oligo against each of the three genomes. For oligos that target ORFs in more than one genome, BLASTN E-values for the multiple target ORFs are always lower than 10^-7 ^and nucleotide percent identities are always greater than 88%. Carter and co-workers, using *Escherichia coli *multistrain arrays with 70 mers probes, state that an identity greater than 80% by BLASTN search matches with hybridization signal intensity [27].

For microarray hybridization 4 μg of genomic DNA was completely digested using EcoRI and subsequently purified and labeled using the BioPrime^® ^Plus Array CGH Genomic Labeling System kit (Invitrogen, Carlsbad, CA) according to the manufacturer's instructions. Control of DNA fragment size distribution and quantity after enzyme digestion and labeling was performed using the Bioanalyzer 2100 (Agilent Technologies, Palo Alto, CA) and the ND-1000 (NanoDrop Technologies, Wilmington, DE).

Microarray processing consisted of hybridization, washing and scanning. Microarray hybridization and washing were performed according to the protocols recommended by The Pathogen Functional Genomic Resource Center [28]. G2534-60001 microarray hybridization chambers were used (Agilent Technologies, Palo Alto, CA). The hybridization solution was a mixture of the two differentially labeled probes. Microarrays were scanned using the Agilent G2565BA scanner.

Four replicates were done for each different pair of test strain and control, resulting in a total of 16 hybridizations. The four different hybridizations were R vs T, R vs Mix (T+R+G), G vs T and G vs Mix (T+R+G). Dye swaps were applied in each set of four replicates so that two had the test sample in the red channel and the other two in the green channel.

Microarray hybridization raw data was deposited in the ArrayExpress [29] public database with the following accession number: E-MEXP-1390.

### Data analysis

Microarray images were analyzed using Feature Extraction 9.1 software (Agilent Technologies, Palo Alto, CA). Spots that were covered by visually identified image artifacts were flagged and discarded. The signal of each spot was background corrected by subtracting the minimum feature signal in the array. A spatial detrend model was fitted to correct for spatial effects. Intensity-specific bias was removed through lowess global normalization. The resulting spot average pixel intensities were used to compute the logarithm (decimal) of the test/control signal ratio, here referred as log-ratios or LR.

The resulting text data files were imported and further processed in the Matlab 7.3 (MathWorks, Natick, MA) computing environment. Gal files provided by The Pathogen Functional Genomic Resource Center [26] containing the gene identification for each spot on the array, as well as information on its presence in each of the three reference genomes were also imported into Matlab. Since each gene was spotted four times per array, data retrieved from each of the valid spots were averaged for a particular gene. Quantile normalization was used to obtain LR distributions of the combined replicate arrays. For a given set of spots within an individual array (where the set can be all spots on the array or subsets as discussed below), and for each possible LR threshold value in that set, we identified each spot as a true positive (TP), true negative (TN), false positive (FP) or false negative (FN). TP spots have a LR greater than or equal to the threshold and target a gene that is present in the test strain. TN spots have a LR lower than the threshold and target a gene not present in the test strain. FP spots have a LR greater than or equal to the threshold and target a gene that is not present in the test strain. FN spots have a LR lower than the threshold and target a gene that is present in the test strain. With the number of TP, TN, FP and FN we can calculate the accuracy (Acc), sensitivity (Sn) and specificity (Sp) according to the following formulas:

$$Acc=\frac{TP+TN}{TP+TN+FP+FN}$$

$$Sn=\frac{TP}{TP+FN}$$

$$Sp=\frac{TN}{TN+FP}$$

The LR threshold producing the maximum Acc value was chosen as the optimal threshold for the corresponding set of spots.

ROC curves were produced by plotting Sn versus 1-Sp values for each possible LR threshold and the area under the ROC curve (AUC) was then estimated for each array or class of spots in each array, which is a measure of the discriminatory power of LR distributions independently of the threshold choice [23].

For both types of control sample we evaluated LR discriminatory power considering all spots together or divided by classes. When the T control is used there are two classes of spots with different control signals (T, TR, TG and TRG spots form one class and R, G and RG form the other class, Table 1). When the mix control is used, there are three classes of spots with different control signals because they target genes present in one control strain (T, R and G spots), two control strains (TR, TG and RG spots) or all control strains (TRG spots). The latter class, constituted by TRG spots, is of no interest for this evaluation because in all the performed hybridizations these spots will have a present call, preventing the setting of a threshold that optimally discriminates between absent and present calls. For this reason, when the spots are analyzed in separate classes, the TRG spots were not considered, both for hybridizations with the T control or with the mix control. For comparison purposes, discarding TRG spots is not anticipated to bias the results since both control types are expected to behave similarly in relation to this class of spots, producing signals of equivalent intensity, eventually with smaller variance for the mix control due to the pooling effect.

To explore differences in AUC, Acc, Sn and Sp values between hybridizations with the T control or the Mix as control, eliminating the effect of different test samples (R or G), we applied the nonparametric Friedman's test [30] and corrected for multiple testing by controlling the False Discovery Rate under or equal to 0.05 through the linear procedure of Benjamini and Hochberg [31].

## Abbreviations

LR – Log Ratio, R – R6, T – TIGR4, G – G54, TR – genes or spots present in TIGR4 and R6, TG – genes or spots present in TIGR4 and G54, RG – genes or spots present in R6 and G54, TRG – genes or spots present in all reference strains, ROC – receiver operating characteristic, AUC – area under the curve, TP – true positive, FP – false positive, TN – true negative, FN – false negative, Sn – sensitivity, Sp – specificity, Acc – accuracy, aCGH – array based comparative genomic hybridization.

## Authors' contributions

FRP, SIA, JM–C and MR conceived the study. SIA performed the experiments and FRP analyzed the datasets generated. All authors wrote, revised and approved the final manuscript.

## Supplementary Material

Additional file 1 LR thresholds. Supplementary Table with LR thresholds producing maximum Acc for the 16 array hybridizations. Click here for file
