# Supplementary material for: Optimal control and analysis of two-color genomotyping experiments using bacterial multistrain arrays
Source: BMC Genomics. 2008 May 19;9:230. doi: 10.1186/1471-2164-9-230 (PMC2410139; doi:10.1186/1471-2164-9-230)
Supplement: Additional file 1 — LR thresholds. Supplementary Table with LR thresholds producing maximum Acc for the 16 array hybridizations. [file 1471-2164-9-230-S1.pdf]

Supplementary Table – LR thresholds producing maximum Acc for the 16 array hybridizations, both when all spots in the array are analyzed together (1 class thresholds in the first column) and when spots are divided into two classes according to expected control signal (2 classes thresholds in the second and third columns). Every unique LR value in a given array (or in a given subset of the array in the 2 classes analysis) is used as a putative threshold, classifying genes with higher LR values as present and the remaining ones as absent. The accuracies of these classifications are computed and the chosen threshold is the one producing maximum accuracy.

	1 class thresholds	2 classes thresholds	
		(T, TR and TG)	(R, G and RG)
RT	-0.1562	-0.5977	0.0524
	0.1683	-0.3321	0.0626
	-0.0981	-0.3353	0.0157
	0.0117	-0.3133	-0.1167
GT	-1.3637	-0.1742	-0.2468
	-0.1373	-0.2005	-0.9085
	-0.0144	-0.0319	-0.0644
	-0.1624	0.035	-1.2927
		(T, R and G)	(TR, TG and RG)
RM	-0.1245	0.1716	0.0299
	-0.0473	0.1964	0.1288
	0.1016	-0.0759	-0.164
	0.2744	-0.0991	0.125
GM	-1.4141	-0.5999	-1.0044
	0.259	-0.2996	0.0188
	0.4837	-0.2285	0.0265
	-1.2745	-0.0289	0.0556
